# A standardized accelerometry method for characterizing tremor: Application and validation in an ageing population with postural and action tremor

**DOI:** 10.3389/fninf.2022.878279

**Published:** 2022-08-04

**Authors:** Etienne Gauthier-Lafreniere, Meshal Aljassar, Vladimir V. Rymar, John Milton, Abbas F. Sadikot

**Affiliations:** ^1^Department of Neurology and Neurosurgery, Montreal Neurological Institute, McGill University Health Centre, McGill University, Montreal, QC, Canada; ^2^Department of Psychiatry, Montreal Neurological Institute, McGill University Health Centre, McGill University, Montreal, QC, Canada; ^3^W.M. Keck Science Department, Claremont Colleges, Claremont, CA, United States

**Keywords:** accelerometry, clinical rating scale, power spectrum, Essential Tremor, sensor, Parkinson, thalamotomy, focused ultrasound (MRgFUS)

## Abstract

**Background:**

Ordinal scales based on qualitative observation are the mainstay in the clinical assessment of tremor, but are limited by inter-rater reliability, measurement precision, range, and ceiling effects. Quantitative tremor evaluation is well-developed in research, but clinical application has lagged, in part due to cumbersome mathematical application and lack of established standards.

**Objectives:**

To develop a novel method for evaluating tremor that integrates a standardized clinical exam, wrist-watch accelerometers, and a software framework for data analysis that does not require advanced mathematical or computing skills. The utility of the method was tested in a sequential cohort of patients with predominant postural and action tremor presenting to a specialized surgical clinic with the presumptive diagnosis of Essential Tremor (ET).

**Methods:**

Wristwatch accelerometry was integrated with a standardized clinical exam. A MATLAB application was developed for automated data analysis and graphical representation of tremor. Measures from the power spectrum of acceleration of tremor in different upper limb postures were derived in 25 consecutive patients. The linear results from accelerometry were correlated with the commonly used non-linear Clinical Rating Scale for Tremor (CRST).

**Results:**

The acceleration power spectrum was reliably produced in all consecutive patients. Tremor frequency was stable in different postures and across patients. Both total and peak power of acceleration during postural conditions correlated well with the CRST. The standardized clinical examination with integrated accelerometry measures was therefore effective at characterizing tremor in a population with predominant postural and action tremor. The protocol is also illustrated on repeated measures in an ET patient who underwent Magnetic Resonance-Guided Focused Ultrasound thalamotomy.

**Conclusion:**

Quantitative assessment of tremor as a continuous variable using wristwatch accelerometry is readily applicable as a clinical tool when integrated with a standardized clinical exam and a user-friendly software framework for analysis. The method is validated for patients with predominant postural and action tremor, and can be adopted for characterizing tremor of different etiologies with dissemination in a wide variety of clinical and research contexts in ageing populations.

## Introduction

Essential Tremor (ET) is a common clinically diagnosed involuntary movement disorder with an estimated prevalence of 0.5% overall and 5% in people over age 65 (Clark and Louis, 2018; Welton et al., 2021). ET can be sporadic or have a familial genetic component (Welton et al., 2021). The frequency of postural limb tremor in ET varies between 4 and 12 Hz and is typically between 4 and 6 Hz. ET is usually bilateral and may have a prominent kinetic component. Rest tremor is more characteristic of Parkinson’s disease. ET can cause significant incapacity especially during fine motor tasks. The pathophysiology of ET remains unclear. Tremor in ET syndromes may result from abnormal cerebellar output associated with structural and functional changes in cerebellar Purkinje cells or neighboring interneurons, deep cerebellar nuclei, the brainstem including the inferior olive, or olivo-cerebellar connecting tracts (Axelrad et al., 2008; Paris-Robidas et al., 2012; Louis and Faust, 2020; Wu et al., 2021; Pan and Kuo, 2022), Other work fails to demonstrate consistent morphological changes (Symanski et al., 2014; Rajput et al., 2019). Ultimately, the abnormal tremor rhythms in ET implicate altered cerebello-thalamo-cortical-spinal circuits and can be suppressed using lesions in the motor thalamus (Louis and Faust, 2020), or pharmacological modulation, including GABAergic or noradrenergic modulators (Ferreira et al., 2019). Whether ET with its predominant postural and action tremor represents a distinct disease entity or is more a syndrome with heterogeneous etiology remains debated in the absence of large definitive clinicopathological correlative studies (Erro et al., 2022). Systematic application of objective, standardized and readily applicable measures of tremor are required for better definition of the postural and action tremor spectrum, and ultimately more effective evaluation of new treatments.

In the research setting, an important initial step to characterizing an oscillation such as in ET is to measure its power spectrum (Milton and Ohira, 2021). The power spectrum provides a quantitative measure of the power in an oscillation as a function of frequency. In contrast to visual ordinal scales used by health care workers to qualitatively rate tremor severity and its impact on quality of life, power spectra derived from sensors provide more objective data with continuous variables (Grimaldi and Manto, 2010; Elble and McNames, 2016). For example, power spectra of tremor derived from accelerometers may be useful for monitoring the progress of individual patients, and for evaluating the response of patient populations to drug therapies, deep brain stimulation, or other novel surgical therapies such as thalamic lesions using Magnetic Resonance Imaging guided Focused Ultrasound (MRgFUS) (Duval et al., 2000; Nistico et al., 2016; van Brummelen et al., 2020; Cernera et al., 2021; Daneault et al., 2021; Erro et al., 2022).

A roadblock for the widespread use of the power spectrum in evaluation of tremor in clinical practice is the lack of reliable and standardized methods for acquisition and analysis. For example, results may vary with the anatomical site of transducer placement, type of sensor, whether acquisition is done in a structured clinical exam setting, duration of sampling, potential fluctuations during longer-term spontaneous recording, or variations in spectral analysis (Mostile et al., 2012; Haubenberger et al., 2016; Schuhmayer et al., 2017; Erro et al., 2022). Here, we present a method that integrates accelerometry with a standardized clinical assessment protocol. Accelerometer wristwatches are used to record upper limb tremor while assuming different postures as part of a structured physical exam similar to that used during the clinical evaluation of tremor. The standardized protocol provides robust and repeatable tremor recordings that are processed with a custom automated analysis framework that includes a user-friendly interface written in MATLAB. The application enables the user to characterize tremor associated with each limb position and is readily integrated into a clinical evaluation. The method yields the acceleration power spectrum of the tremor recording in standard arm postures, and also provides the user with measures of peak frequency, peak power, and total power of the acceleration power spectrum. Signal processing is performed with an intuitive graphical interface that can be adapted to the user’s specific needs. The resulting data can be summarized graphically and archived for individual or pooled analyses. The interface is designed to allow efficient application with modest training by a wide variety of users, including specialized researchers, clinicians or allied health personnel who may have little or no programming experience in MATLAB.

The presented method for tremor analysis is applied prospectively in a sequential cohort of patients with postural and action tremor diagnosed with ET in specialized movement disorders clinics and evaluated for surgical treatment. The method is also validated using concomitant evaluation with the commonly used Clinical Rating Scale for Tremor (CRST) (Fahn et al., 1993; Stacy et al., 2007; Ondo et al., 2018). We demonstrate that the standardized accelerometry method provides valid measures of ET, detecting the predominant postural and action components, and distinguishing from rest tremor. The measures also correlate well with CRST scores. The accompanying power spectra from accelerometry provide more precise quantitative estimates of each patient’s tremor frequency and severity. To further illustrate clinical application, we demonstrate the utility of the structured protocol in evaluating changes in an ET patient treated with MRgFUS thalamotomy. We propose that integrating wristwatch accelerometry, a standardized clinical exam and a user-friendly software framework provides useful quantitative measures of tremor with results presented as a continuous variable over a broad range. The method can be readily adapted for characterizing tremor of different etiologies in ageing patients, and applied in a wide variety of clinical and research contexts.

## Materials and methods

### Participant demographics

Twenty-five patients (23 right-handed, 2 left-handed; 7 females, 18 males; mean age 70.5 ± 6.3, SD) with predominant postural and action tremors were diagnosed with Essential Tremor (ET) in specialized movement disorders clinics. The patients were evaluated sequentially for surgical treatment at the Montreal Neurological Institute and included in the protocol. A control group of six male volunteers with no movement disorders in a matching age range as patients also participated. An illustrative case example of surgical treatment response and longitudinal follow-up of tremor is also presented. All participants provided written consent and were part of a research protocol approved by the McGill University Health Center’s Research Ethics Board.

### Acquisition protocol

Laboratory grade wristwatches with micro-electromechanical systems (MEMS)-based accelerometers that are widely applied in research (GENEActiv^©^ Original, Activinsights™, United Kingdom) were used (Pavey et al., 2016; Sanders et al., 2019; Daneault et al., 2021). They record data continuously (up to 100 Hz; ±8 Gs acceleration sensing range; 3.9 mG resolution; 0.5 Gb non-volatile memory), are waterproof, rechargeable and equipped with a light-activated silicone photodiode. The accelerometer data was extracted in bin format and converted into cvs tables using GENEActiv^©^ software.

Participants were off ET-related medication, caffeine and alcohol for 12 h, and removed upper limb jewelry. The CRST and accelerometry were performed concomitantly to provide time-matched comparisons. Data acquisition and analysis were performed by different investigators, allowing blinded analysis.

For the accelerometry acquisition, two GENEActiv^©^ Original watches were secured to either wrist (Supplementary Figure 1). The structured clinical examination protocol began with the patient seated comfortably in an armchair. The prescribed positions were rehearsed with the patient prior to the recording, and active instructions were given during the recording. The exam includes: (1) Rest Position: The participant’s elbows were flexed, and both forearms were pronated and rested comfortably fully supported on padded armrests. (2) Postural Position: One arm was extended in front of the participant parallel to the floor, with the forearm pronated and fingers extended. The contralateral arm stayed in the rest position. These measures were repeated in the contralateral limb. (3) Postural Position, Bimanual: Both arms were extended. (4) Wing Position, Bimanual: Both arms were abducted at the shoulder, the elbows were flexed and at the same height as the shoulders, with the fingers extended and about an inch apart in front of the chest. (5) Action with Loading: One arm extended fully in front close to a target while the participant held a closed plastic 500 mL water-filled bottle, and performed a to-and-fro motion from the mouth to the target approximately once per second, mimicking a drinking action. The contralateral arm remained in the rest position. Each position was held for 30 s, with intervening 30 s rest periods. About 5–15 s of buffer time was provided between each position to allow comfortable changes and to account for re-emergent tremor during postural conditions. A flashlight activated a light sensor in the wristwatch at the start and end of each tremor epoch to allow reliable assignment of positions during analysis (Supplementary Figures 1, 4).

The protocol was repeated 3× to allow for three sampling epochs per active position with 12 rest position epochs. The entire recording session with setup time lasts ∼30 min. The data was downloaded to a computer with GENEActiv^®^ software. To allow the app to automatically process useful recording information, the patient identifier, wrist side, watch number, date and time of recording were included in the filename, separated by underscores as in the following example: *patient X_right wrist_watchnumber_2018-07-13 17-27-44.csv*.

Each patient’s tremor was assessed by a qualified physician during the same session using the Clinical Rating Scale for Tremor (CRST) (Fahn et al., 1993; Elias et al., 2016). The CRST rater was blind to the accelerometry results. Although the CRST does not evaluate tremor in the wing or bimanual positions, they were included in the accelerometry measurement protocol.

### Accelerometry analysis

The supplementary section details the method developed for tremor analysis. The narrative includes a description of acquisition of raw data, coding and filtering, sorting of relevant epochs used to generate power spectra, principal component analysis of the acceleration signal, and Fourier analysis to generate the power spectrum of tremor acceleration. In addition, Supplementary Figures 1–3 illustrate the acquisition protocol (Supplementary Figure 1), conceptual diagram of analysis workflow (Supplementary Figure 2), the software platform and graphical user interface (Supplementary Figure 3), and final tremor epochs selected for analysis (Supplementary Figure 4). A graphical comparison of accelerometry results in ET and control groups (Supplementary Figure 5) is provided. Finally, graphical examples are given of three patients with a range of CRST scores and their associated power spectra (Supplementary Figure 6).

## Results

**FIGURE 1:**
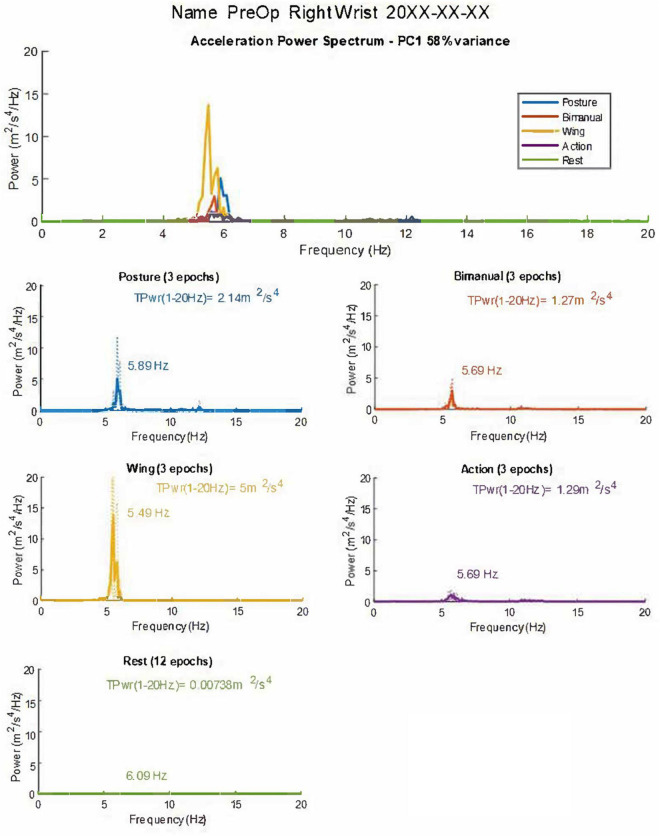
Graphical summary of tremor accelerometry analysis as presented to the user. The combined and individual power spectra for the different postures are shown. Dotted lines represent individual epochs, and full lines represent the average of the three epochs. The first principal component of the accelerometry recording (PC1-58% of the variance) is used to derive and plot measures. TPwr = Total power of acceleration in the 1–20 Hz band. Frequency and peak power are also provided in graphs. In this case, the dominant frequency of tremor in the acceleration spectrum is mainly stable in different postures, with a slight reduction in the Wing position (see Table 1). TPwr varies significantly, increasing in postural conditions with the highest power from accelerometry at the wrist in the Wing position. The patient’s name and date of the recording are anonymized for publication.

All 25 consecutive Essential Tremor (ET) patients successfully completed the structured protocol and useful accelerometry data was acquired in all cases. Overall, average peak tremor frequency across all positions was 4.57 ± 1.8 Hz, SD. Tremor characteristics in each upper extremity position assumed by patients as part of the structured physical examination were analysed separately (Figure 1 and Tables 1–3). The average peak frequency was similar in the postural conditions (extended limb, unimanual, 4.78 ± 1.23 Hz, SD; extended limb, bimanual, 4.89 ± 1.43 Hz; wing position, 4.58 ± 1.35 Hz). During action, mean peak frequency (3.81 ± 2.02 Hz, SD) was lower, but the difference was not statistically significant (Table 1). Frequencies detected at rest (4.78 ± 3.03 Hz, SD) were highly variable as reflected in the standard deviation, and analysis of power spectra indicated far lower values of total and peak power at rest compared to postural and action conditions (Table 1 and Supplementary Figure 5), as detailed further below.

**TABLE 1:** Summary of accelerometry measures.

	Peak frequency		Peak power		Total power (1–20 Hz)	
Position	Hz		m^2^/s^4^/Hz		m^2^/s^4^	
	Mean	SD	Mean	SD	Mean	SD
Rest	4.78	3.03	0.02	0.05	0.11	0.31
Posture	4.78	1.23	1.58	4.33	1.08	2.62
Bimanual	4.89	1.43	1.44	3.47	0.96	2.18
Wing	4.58	1.35	8.37	18.7	1.9	3.94
Action	3.81	2.02	1.33	1.46	1.88	3.67

The mean peak frequencies in the different postural positions were similar, with minor reduction in the action position that was not statistically significant. Peak power increases ∼60–80 fold in postural and action conditions compared to rest. Peak power increases ∼5 fold in the wing position compared to the arm extended postural condition. Total power increases ∼10 fold in postural conditions (unimanual and bimanual) compared to rest. There is a ∼2 fold increase in total power in the wing and action conditions compared to the arm extended postural condition. SD, Standard Deviation. Power at rest is markedly low compared to postural and action conditions.

**TABLE 2:** Correlations of CRST (Part A) scores with the total power of acceleration (m^2^/s^4^) at the wrist in the dominant arm in the different rest, postural and action conditions.

	Rest			Posture			Action			Bimanual			Wing		
CRST Part A	*r*_*s*_	95% Cl	*P*-value	*r*_*s*_	95% Cl	*P*-value	*r*_*s*_	95% Cl	*P*-value	*r*_*s*_	95% Cl	*P*-value	*r*_*s*_	95% Cl	*P*-value
Rest	0.352	−0.062–0.662	0.0847	0.238	−0.186–0.586	0.2525	0.368	−0.044–0.673	0.0703	**0.439**	0.040–0.716	0.0281*	0.266	−0.156–0.606	0.1991
Posture	0.121	−0.298–0.502	0.5635	**0.489**	0.104–0.746	0.013*	**0.588**	0.239–0.802	0.002**	**0.601**	0.258–0.809	0.0015**	**0.493**	0.108–0.748	0.0123*
Action	0.367	−0.045–0.672	0.0710	**0.601**	0.258–0.809	0.0015**	**0.689**	0.393–0.855	0.0001***	**0.567**	0.210–0.790	0.0031**	**0.490**	0.105–0.747	0.0129*
Total	0.373	−0.038–0.676	0.0662	**0.658**	0.343–0.839	0.0004***	**0.779**	0.545–0.900	< 0.0001****	**0.765**	0.521–0.893	< 0.0001****	**0.609**	0.269–0.813	0.0012**

Total power of acceleration in two postural conditions showed moderate (Postural, Unimanual) or strong (Postural, Bimanual) and significant correlations with the corresponding postural CRST scores. Total power of acceleration in the action condition showed strong significant correlation with the corresponding action CRST score. Total power of acceleration in both postural and action conditions showed strong and significant correlations with the corresponding total CRST scores. Results from the wing position were presented as part of the correlation table, but this position was not part of the CRST. Bold font signifies moderate and strong correlations. r_*s*_ is the Spearman correlation coefficient, CI is the confidence interval, the *P*-value is two tailed, *P < 0.05, **P < 0.01, ***P < 0.001, ****P < 0.0001.

**TABLE 3:** Correlations of CRST (Part A) scores with the peak power of acceleration (m^2^/s^4^) at the wrist in the dominant arm in the different rest, postural and action conditions.

	Rest			Posture			Action			Bimanual			Wing		
CRST Part A	*r*_*s*_	95% Cl	*P*-value	*r*_*s*_	95% Cl	*P*-value	*r*_*s*_	95% Cl	*P*-value	*r*_*s*_	95% Cl	*P*-value	*r*_*s*_	95% Cl	*P*-value
Rest	0.325	−0.093–0.645	0.1134	0.327	−0.090–0.646	0.1107	**0.413**	0.009–0.701	0.04*	**0.512**	0.134–0.759	0.0089**	0.357	−0.056–0.666	0.0795
Posture	0.167	−0.255–0.536	0.4242	**0.616**	0.280–0.817	0.001**	**0.628**	0.298–0.823	0.0008***	**0.691**	0.395–0.856	0.0001***	**0.564**	0.205–0.789	0.0033**
Action	0.388	−0.020–0.685	0.0552	**0.480**	0.093–0.741	0.0151*	**0.576**	0.221–0.795	0.0026**	**0.477**	0.088–0.739	0.016*	**0.429**	0.028–0.710	0.0325*
Total	0.396	−0.011–0.690	0.0500	**0.668**	0.360–0.844	0.0003***	**0.758**	0.508–0.889	0.0001***	**0.775**	0.538–0.898	< 0.0001****	**0.638**	0.313–0.829	0.0006***

Peak power of acceleration in two postural conditions (Postural, Unimanual; Postural, Bimanual) showed strong and significant correlation with the corresponding postural CRST scores. Peak power of acceleration in the action condition, showed moderate and significant correlation with the corresponding action CRST score. Peak power of acceleration in both postural and action conditions showed strong and significant correlations with the corresponding total CRST scores. Results from the wing position are presented as part of the correlation table, but this position is not part of the CRST. Bold font signifies moderate and strong correlations. r_*s*_ is the Spearman’s correlation coefficient, CI is the confidence interval, the *P*-value is two tailed, *P < 0.05, **P < 0.01, ***P < 0.001, ****P < 0.0001.

In contrast to frequency, the mean total power (1–20 Hz) and peak power in the power spectrum of acceleration showed significant variation across different positions (Table 1, Figure 1 and Supplementary Figures 5, 6). Interestingly, increases were noted in all static postural conditions compared to rest, in keeping with the diagnosis of an ET syndrome. Total and peak power of acceleration was especially accentuated in the wing position. To determine whether the increase in power measured in action was more a reflection of translational artefact due to back-and-forth movement rather than tremor oscillations, control participants (*n* = 6) without clinically detectable tremor performed a similar protocol as ET patients. Mean total power of acceleration in the action with loading condition in controls was 0.09 ± 0.03 m^2^/s^4^, which is 21 times lower than in ET patients, indicating that the high level of total power during action in ET patients was mainly a reflection of tremor detected by the accelerometer-based analysis protocol rather than movement alone (Supplementary Figure 5). Furthermore, control participants showed peak and total power that was also many magnitudes below that seen in the patients (Supplementary Figure 5).

To determine how measures of total power within the 1–20 Hz band and peak power compare with a commonly used clinical scale, CRST upper extremity tremor scores were obtained during the same session as accelerometry, as part of the structured protocol. Systematic Spearman’s correlations were performed between CRST scores and accelerometry, using measures from the dominant arm. Overall, significant moderate to strong correlations were obtained in postural and action conditions between accelerometry measures and the CRST Part A, which provides a visual assessment of upper extremity tremor (Tables 2, 3). The CRST Part A showed significant moderate to strong correlations with total power of acceleration in both postural conditions (Postural, Unimanual, *r*_*s*_ = 0.489, *p* = 0.013^∗^; Postural, Bimanual, *r*_*s*_ = 0.601, *p* = 0.0015^∗∗^, see Table 2). Peak power of acceleration showed strong significant correlation with CRST Part A scores in both postural conditions (Postural, Unimanual, *r*_*s*_ = 0.616, *p* = 0.001^∗∗^; Postural, Bimanual, *r*_*s*_ = 0.691, *p* = 0.0001^∗∗∗^, see Table 3).

Furthermore, in the action condition, the CRST Part A showed strong significant correlation with total power of acceleration (*r*_*s*_ = 0.6888, *p* = 0.0001^∗∗∗^, Table 2) and a moderate significant correlation with peak of acceleration (*r*_*s*_ = 0.5756, *p* = 0.0026^∗∗^, Table 3). The total CRST part A score showed strong and significant correlations with total and peak power of acceleration in both postural and action conditions as expected for this group of ET patients. Results from the wing position are presented as part of the correlation tables (Tables 2, 3), but this position is not part of the CRST. However, it does appear in other visual clinical scales for ET, including the TETRAS.

Rest tremor was not significant in the majority of patients (Table 1 and Supplementary Figure 5), with expected non-significant weak correlations between CRST Part A scores and power of acceleration. We noted peak power of tremor at rest above the evidently low mean (0.024 m^2^/sec^4^ ± 0.047, SD) in five patients. In 4 of these 5 cases, the ratio of rest peak power/postural peak power was much less than 1, and in one case it was 1.03. This one patient had the third highest peak power in action, with comparatively low power at rest and during postural conditions. Four patients had total power at rest above the evidently low mean (0.11 m^2^/sec^4^ ± 0.31, SD), and these cases were within the group of five patients with peak tremor above the mean. Of these, two had a ratio of rest total power/postural total power above 1, and both these cases had high power in action and were classed in the top four in the group of 25. These patients with advanced ET may have been considered as having an “ET plus” variant according to a recent consensus classification (Bhatia et al., 2018) although they may also be considered as a variant of advanced ET (Louis et al., 2020; Iglesias-Hernandez et al., 2021).

The range of power spectrum outputs typified by different severity of tremor from CRST (Part A) scores, is further illustrated (Supplementary Figure 6). We noted the wide range of values captured by accelerometry for different CRST ratings, as evident in variations in the magnitude of the *y*-axes. Thus, power in Example 1, with a low CRST score, is many orders of magnitude smaller than values in Example 3, with a high CRST score. A large measurement range is afforded by accelerometry, and contrasts with the relatively low range of visual scales that limits the appreciation of differences in tremor severity between patients.

**FIGURE 2:**
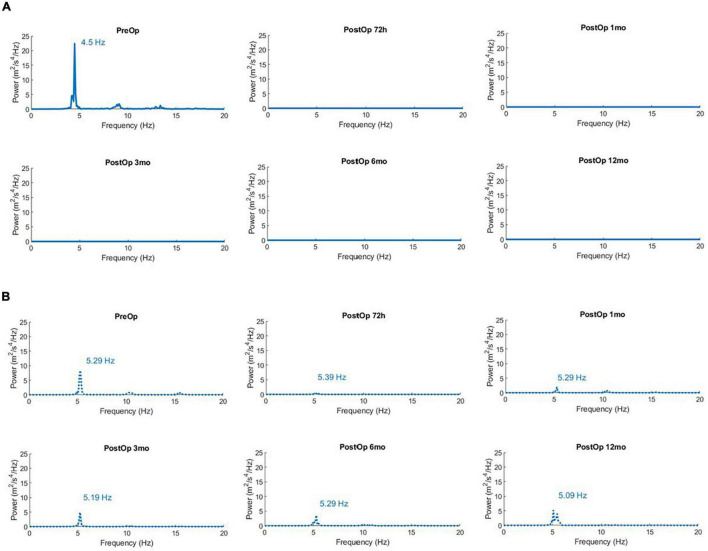
**(A)** Power Spectra of Acceleration for the right arm (dominant) in the unimanual extended position (Posture) showing successful reduction of tremor after left brain MR-guided focused ultrasound thalamotomy. Time points are prior to surgery (PreOp), and at five intervals after surgery (PostOp). The total power of acceleration in the 1–20 Hz band is 7.1 m^2^/s^4^ prior to surgery, and consistently < 0.01 m^2^/s^4^ in subsequent measures spanning 1 year after surgery. **(B)** Power Spectra of Acceleration for the non-operated left arm in the unimanual extended position (Posture) after MR-guided focused ultrasound thalamotomy. The total power of acceleration in the 1–20 Hz band is 3.51 m^2^/s^4^ prior to surgery, and then 0.197, 0.906, 1.44, 1.79, and 2.72 m^2^/s^4^ in subsequent measures spanning 1 year after surgery.

To further illustrate the practical utility of an integrated standardized physical exam and accelerometry protocol, we describe the case of a patient who underwent MR-guided Focused Ultrasound Thalamotomy (MRgFUS), an emerging novel treatment for the control of refractory ET (Figure 2). A 76-year-old right-handed male presented with a 25-year history of tremor in both arms. His tremor had worsened during the last 5 years. The tremor severely impaired daily activities such as writing, eating, drinking and dressing, and was resistant to multiple attempts at pharmacological treatment. Tremor in the dominant right arm was treated with a MR-guided Focused Ultrasound lesion in the left thalamic ventral intermediate nucleus (Vim) (Atkinson et al., 2002; Su et al., 2020). The structured accelerometry protocol and CRST scale were performed prior to surgery and at each clinical follow-up at 48 h and 1, 3, 6, 12 months after treatment (Figure 2). The patient’s right arm tremor was more disabling and showed higher total power. The tremor power spectrum was silenced post-operatively at the right arm, with no re-emergence on serial evaluations up to 1 year after treatment (Figure 2). The patient experienced marked improvement in activities of daily living (eating, drinking from a glass, dressing) and gradually stopped medications for control of tremor. This method can therefore provide valuable information on tremor frequency, tremor power, effects of different postures, and effects of medical interventions on tremor severity that can be followed over time.

## Discussion

Tremor as a clinical finding is commonly defined as a rhythmic shaking of a body part (Clark and Louis, 2018; Welton et al., 2021). Involuntary tremor is generally composed of non-linear, non-stationary roughly sinusoidal oscillatory movements with a symmetric velocity profile about a theoretical midpoint (Grimaldi and Manto, 2013; Deuschl et al., 2022). Pathological tremor can produce significant functional disability, limit daily activities and contribute to social withdrawal (Thangavelu et al., 2020). A wide variety of tremor syndromes can be distinguished by clinical history, associated neurological features, and involvement of different body parts. Physical examination of tremor at rest or with different postural or kinetic conditions allows for visual approximation of tremor frequency and amplitude, and is the mainstay for diagnosis and treatment follow-up (Bhatia et al., 2018; Welton et al., 2021). Common pathological tremors include Essential Tremor (ET) (4–12 Hz), other types of cerebellum related tremors (2–10 Hz) including the irregular and lower frequency Holmes tremor (3–5 Hz), rest tremor typically seen in Parkinson’s disease (3–6 Hz), and the higher frequency orthostatic lower limb tremor (13–18 Hz) (Grimaldi and Manto, 2013; Ondo et al., 2020; Deuschl et al., 2022).

One of several visual scales is used for systematic evaluation of tremor in ET, including the Fahn-Tolosa-Marin Tremor Rating Scale (FTM-TRS) (Fahn et al., 1993) or Clinical Rating Scale for Tremor (CRST) (Stacy et al., 2007; Elias et al., 2016), the more recent Tremor Research Group Essential Tremor Rating Assessment Scale (TETRAS) (Elble et al., 2012; Ondo et al., 2018; Tarakad, 2020), the Bain and Findley Clinical Tremor Rating Scale (BF-TRS) and the Glass Scale (Tarakad, 2020). Since tremor is a continuous variable, ordinal scales are limited by non-linearity, and ceiling and floor effects. Tremor amplitude is not perceived linearly by raters, but rather follows a logarithmic relationship when compared to more objective measures like accelerometry (Elble et al., 2006; Elble, 2018). The widely used CRST shows very good internal consistency but suffers from moderate to fair inter-rater reliability, and its 0–4 scale of tremor amplitude is susceptible to ceiling effects (Elble et al., 2012; Lee et al., 2015). The TETRAS is less susceptible to ceiling effects since it assigns a wider range of tremor amplitudes and shows very good inter-rater reliability (Elble et al., 2012; Ondo et al., 2018). The TETRAS also includes the wing posture unlike the CRST, but it requires more training and does not include a rest position (Elble et al., 2012). Like other visual scales it fails to adequately capture frequency, does not provide a continuous variable, and cannot provide a power spectrum of acceleration. These limitations motivate the development of standardized digital methods that directly quantify tremor displacement or acceleration (Grimaldi and Manto, 2010; Elble, 2018; Fitzgerald et al., 2018). Such standardized quantitative measures of tremor can prove useful for individual patients, and can be archived for analysis in larger-scale clinical trials or longitudinal studies involving multiple evaluators.

A variety of effective but technically intensive methods are described for quantitative evaluation of tremor. These include electromyograms that assess changes in voltage over active muscle groups as a surrogate for tremor amplitude and frequency (Breit et al., 2008; Zhang et al., 2017), electromagnetic motion capture (Charles et al., 2017; Geiger et al., 2018), laser-based displacement sensors (Duval et al., 2000) and wearable devices based on accelerometry (Hossen et al., 2013; Luft et al., 2019) or gyroscopic measurements of angular speed (Jeon et al., 2017; Berbakov et al., 2019). Gyroscopes may be more appropriate compared to accelerometers when measuring movements with a significant angular velocity component such as head tremors (Elble et al., 2017). Laser-based displacement sensors provide direct measures of tremor amplitude, in contrast to accelerometry or gyroscopic measures, that require derivative calculations to yield amplitude from acceleration. However, laser displacement sensors are limited by portability (Duval et al., 2000). They are difficult to integrate with the clinical exam and cannot readily measure action tremor. In some cases, multiple modalities are successfully applied, such as combinations of accelerometry and electromyography (Ferreira et al., 2019; Luft et al., 2019; Shanker, 2019). These sophisticated quantitative methods, while effective, are generally not translated from research settings to clinical applications (Fitzgerald et al., 2018). Accelerometry has gained popularity over other sensors with availability of portable micro-electro-mechanical systems (MEMS) that integrate small transducers with microelectronic circuits for data acquisition and storage (Elble and McNames, 2016). These sensors allow for the acquisition of quantitative data that include power spectra of acceleration, frequency and derivative measures of amplitude. These measures are advantageous compared to more subjective, non-linear visually guided clinical ratings scales. Despite ready availability, wider dissemination of accelerometers in clinical settings is impaired by a relative paucity of validation in different tremor types, lack of prescribed standardized positions for evaluation of tremor in different body parts, lack of user-friendly freely available analysis software and lack of standards for the wide variety of available transducers (Elble and McNames, 2016; Haubenberger et al., 2016; Erro et al., 2022). Furthermore tremor is posture dependent, varies in different body parts, and fluctuates over time (Cleeves and Findley, 1987; Mostile et al., 2012; Schuhmayer et al., 2017). Thus, there is a need for standardized protocols that effectively integrate accelerometry with the clinical exam, preferably with simultaneous acquisition of a clinical rating scale.

Applications that measure upper extremity tremor with a smartphone that contains accelerometers or gyroscopes and is held in the palm of the hand (Daneault et al., 2012; Lopez-Blanco et al., 2018) are cost-effective and readily available. However, wider application is restricted by differences in smartphone brands, the proprietary nature of the hardware and analysis software, lack of standardized evaluation protocols and difficulty adapting handheld devices to conventional clinical exams. Furthermore, tremor characteristics may be modified by the mass and grasp of the handheld device and limit generalizability (Daneault et al., 2012; Pulliam et al., 2014; Wile et al., 2014; van Brummelen et al., 2020). In contrast, smart-watches do not add a significant weight-loading element, are more readily adapted to the neurological exam, and can serve as a wearable for longer-term evaluation. Analysis of tremor of the upper limb in each of the seven degrees of freedom identifies the wrist as holding the greatest amount of power associated with ET (Charles et al., 2017; Geiger et al., 2018), and allows capture of most of the tremor power during the different positions used in a structured clinical exam. Barriers to wider application of wristwatch accelerometers include lack of standardized administration protocols, cumbersome data analysis and sparse validation in clinical practice (Elble and McNames, 2016). When considering accelerometry as an adjunct to clinical scales, it is important to consider fluctuation of tremor over time (Cleeves and Findley, 1987; Pulliam et al., 2014). It is therefore more useful to integrate both the accelerometry and standardized clinical scale assessment during the same session. Here, we incorporated accelerometric measurements into a standardized protocol, with positions generally prescribed during clinical evaluation of tremor, including measures at rest, three different postures (outstretched arm unilateral or bilateral, wing) and action (van de Wardt et al., 2020). The protocol allowed for efficient accelerometry measurements at the same time as the CRST and can be adapted to other preferred clinical scale assessments used for ET syndromes or other upper limb tremor syndromes.

A variety of affordable consumer grade accelerometer watches provide high sampling rates over sufficient intervals. The watches used in the present study can be set to sampling rates up to 100 Hz with a resolution of 3.8 mG providing adequate precision for digital tremor analysis. While tri-axial acceleration measurements do not provide direct evaluation of tremor amplitude, in contrast to other tools such as laser displacement sensors (Duval et al., 2000; Carignan et al., 2012), they provide convenient and useful derivative measures of tremor applicable to diagnosis and research. The acceleration power spectrum enables identification of the peak tremor frequency. This information can contribute to diagnosis by distinguishing lower frequency tremors from high frequency tremors, or in some cases support the diagnosis of a functional tremor when there is no consistent peak (Grimaldi and Manto, 2013; Louis, 2013; Hallett, 2016; Deuschl et al., 2022). The total power of acceleration and peak power are effective quantitative measures of tremor severity and may be used as an archive for follow-up studies of tremor progression and treatment efficacy (Elble et al., 2006; Grimaldi and Manto, 2013; Hossen et al., 2013; Charles et al., 2017; Lopez-Blanco et al., 2018; Luft et al., 2019; van Brummelen et al., 2020).

The integrated accelerometry and structured physical exam protocol and custom MATLAB analysis package was successfully implemented in a cohort of clinically well-characterized patients being evaluated for surgery for ET. The method reliably provided tremor frequencies consistent with the diagnosis of ET and identified the predominant tremor postural and action components. The ET cohort had postural (4.78 ± 1.23 Hz, SD) and action (3.81 ± 2.02, SD) tremor at the lower end of the typical reported range of 4–6 Hz (Shanker, 2019). Since tremor frequency tends to decrease and amplitude increases with disease progression (Elble, 2000; Hellwig et al., 2009; Grimaldi and Manto, 2013), the observed frequencies likely reflect the advanced disease state of this medically resistant surgical cohort. Interestingly, tremor frequency is similar in all postural positions, which suggests the predominant influence of central networks as generators with peripheral proprioceptive modulation (Duval et al., 2000; Raethjen and Deuschl, 2012). Tremor amplitude is a major determinant of disability and is reflected in the power spectrum of tremor. Whereas frequency is similar in different positions, the marked increase in power in posture and action conditions compared to rest (Supplementary Figures 5, **6**) is consistent with ET and provides an index of disability. Peak power of tremor correlates best with CRST Part A scores (Table 3), which is expected, since peak power reflects tremor intensity at its dominant frequency. The wing position is included in scales designed for ET such as the TETRAS. The algorithm demonstrated that the wing position enhanced tremor, in keeping with other work with ET patients, especially those that systematically apply the TETRAS (Ondo et al., 2020). Total power of tremor also tends to be higher in the kinetic/action condition compared to when the arm is extended, reflecting clinical observations in ET (Louis, 2013; Ondo et al., 2020).

Overall, the present cohort had low power at rest compared to postural and action tremor. Notably, total power at rest was above the mean in four cases. A recently introduced classification considers ET as a tremor syndrome rather than a disease (Bhatia et al., 2018). An “ET plus” category is added to account for patients with other neurological signs such as impaired tandem gait, possible dystonic posturing, memory impairment, rest tremor or “soft signs” of uncertain clinical significance. Inclusion of a distinct ET plus category for patients with rest tremor has generated some controversy, in part due to previous work suggesting that ET patients can have rest tremor especially in advanced stages and at long duration (Tarakad and Jankovic, 2018; Fearon et al., 2019; Elble, 2020; Louis et al., 2020; Pandey et al., 2020). Indeed, since measures adopted for ET such as the TETRAS do not consider rest tremor, some studies may underestimate its prevalence in ET. Furthermore, the test-retest reliability for rest tremor in patients with “ET plus” is poor (Ondo et al., 2018). In the present cohort, patients with significant rest tremor also had higher values of total power in the action condition, and may represent a more advanced group of ET tremors, or could be classified as “ET plus” (Bhatia et al., 2018; Iglesias-Hernandez et al., 2021).

More objective measures of tremor with sensors can be applied in a manner that is agnostic to “disease” classification or etiology, and help characterize a tremor syndrome, which can then be associated with presumptive disease entities. By incorporating the prescribed accelerometry positions into a standardized clinical exam the present wrist accelerometry method is designed to mitigate variability in sensor output due to differences in application by different observers. The method can integrate accelerometry measures in different upper extremity positions with well-used clinical scales, providing objective and continuous variables that assist in defining tremor-associated phenotypes of patients in the clinic or for research purposes. Characterisation of tremor spectra based on sensors therefore does not rely on fluid categories whose pathological basis has yet to be precisely defined. Additional work on subtypes of the ET syndrome (Elble, 2020; Becktepe et al., 2021; Gionco et al., 2021; Iglesias-Hernandez et al., 2021), with objective characterisation of tremor in different positions and over time, may provide better insight into the ET spectrum. Correlation of these cohorts evaluated with standardized methods at single or multiple centers with brain imaging and eventual post-mortem studies will better define disease entities within the tremor spectrum.

As proof of principle, the evaluation protocol was also applied in a patient with medically intractable ET prior to MRgFUS thalamotomy and during subsequent postoperative follow-up examinations. The patient underwent ventral intermediate nucleus (Vim) thalamotomy (Schaltenbrand et al., 1977), targeting an area within the posterior part of the ventral lateral nucleus (VLp) of the thalamus (Jones, 2007). The standardized accelerometry protocol quantified tremor before surgery, the marked reduction after surgery, and the persistent tremor relief on repeated measurements over a year. The method may be widely applied to determine effectiveness of novel approaches in individuals or groups, including MRgFUS, deep brain stimulation surgery, open thalamotomy or other medical treatments for tremor.

The standardized protocol was readily followed despite language barriers or mild cognitive dysfunction in some patients. The software framework allowed reliable extraction of tremor metrics, including the acceleration power spectrum, peak frequency, peak amplitude, total power of tremor, and graphical tremor profiles. Data extraction requires a computer interface and some technical knowledge that is readily acquired. Several steps would further validate and advance the present methodology. First, inter-rater reliability of the protocol could be compared in different clinical and research domains. This would include refinement of the open-source user-friendly software framework in wider field application by different caregivers, researchers or allied health professionals. Second, heterogeneous tremor types could be tested. Third, the software framework could be tested using data from different accelerometer wristwatches, enabling broader applicability. Fourth, a comparison with other digital acquisition methods such as EMG or laser displacement sensors would be useful, although the portability and ease of use of accelerometer watches is a distinct advantage. Fifth, accelerometer-based methodology may be adapted to other body parts. Finally, while standardized accelerometry with wearable sensors can provide an excellent snapshot of tremor characteristics in controlled environments (Dai et al., 2015; Pan et al., 2015; Delrobaei et al., 2018; López-Blanco et al., 2019), future development may include measures of tremor in diverse environments such as in-home contexts. However, long term task independent monitoring of tremor remains challenging in view of data storage requirements, and a lack of readily available protocols for analysis of tremor in freely moving limbs (Salarian et al., 2007; Pulliam et al., 2018).

In summary, we present a novel accelerometry-based structured protocol that provides objective, reliable, and reproducible measures of upper extremity tremor, distinguishing rest, postural and action components. Accelerometry provides a continuous variable, with measures of frequency and derivatives of the power spectrum that reflect tremor intensity. Since the algorithm combines digital tools with a standardized protocol that mirrors a commonly applied physical examination, it can be readily adapted to the clinic. Systematic use in the clinic will provide a basis for rational interpretation of variations in tremor as part of natural history or in response to novel therapies (Sharma and Pandey, 2020; Erro et al., 2022). The suggested standardized clinical evaluation protocol can be readily integrated with clinical tremor scales (e.g., CRST or TETRAS), providing additional information on the impact of tremor on daily functions such as handwriting, and results of the spiral and line drawings that are especially sensitive to postural and kinetic tremors. Since tremor syndromes are defined using multiple clinical criteria such as onset, evolution, family history, body part involved and possible etiology (Bhatia et al., 2018; Welton et al., 2021), digital assessment tools cannot replace clinical tremor assessment scores and quality of life assessments (Elble et al., 2013; Tarakad, 2020). Rather, they serve as an important adjunct to the clinical exam, providing a comprehensive evaluation of tremor and a useful approach for harmonizing with research-based methods. Future work will determine generalizability of the method for evaluation of other tremor types, including Parkinson’s disease and related conditions.

## Data availability statement

The data that support the findings of this study are available from the corresponding author, upon reasonable request.

## Ethics statement

The studies involving human participants were reviewed and approved by the McGill University Health Centre Institutional Review Board. The patients/participants provided their written informed consent to participate in this study.

## Author contributions

EG-L developed the software, performed the analysis, helped with protocol design, and wrote initial draft of manuscript and participated in writing final version. MA developed clinical evaluation protocol and data acquisition method, reviewed and helped implement software application, helped write initial and final drafts of manuscript, and participated in writing final version. VR helped with statistical analysis, and reviewed final drafts of manuscript. JM reviewed the applied mathematics and data analysis, and helped write final version of manuscript. AS supervised the project, obtained research funding, helped conceive project plan and protocol, helped with participant recruitment, reviewed software development and helped with implementation, helped with data analysis, helped write and supervise final versions of manuscript with input from all collaborators. All authors contributed to the article and approved the submitted version.

## Supplementary material

Supplementary Figure 1 **(A)** Timeline of standardized protocol used to record tremor in different upper limb positions during wristwatch based accelerometry. Data acquisition is performed sequentially at rest, then with with one arm extended (Posture Unimanual), followed by extension of the contralateral arm, bimanual arm extension (Posture Bimanual), bimanual wing position (Wing), and finally with either arm moving with a to and fro drinking motion whilst holding a 500 mL water filled plastic bottle (Action). One or two signals were applied to activate the light sensor on the wristwatch, signaling the start or end of different standardized postural conditions. **(B)** Resulting raw data from the acquisition. Blue, yellow, and orange traces represent detrended x,y,z accelerometry data. Red trace represents luminosity (lux), used as an event marker.

Supplementary Figure 2 Conceptual diagram of the analysis workflow from data acquisition (solid vertical line and bounding boxes) to analysis (chevron-pattern vertical line and interrupted boxes). Watch logo represents steps using GeneActiv^©^ accelerometer watches and accompanying software. Laptop logo represents steps using the customized application created in MatLab, designed for tremor analysis (see Supplementary Figure 3).

Supplementary Figure 3 **(A)** Graphical user interface (GUI) visualized upon launch of the MatLab application (Tremor App). Basic analysis settings (dashed box) can be verified and changed. Advanced settings (dotted box) including features such as filter design and principal component visuals can also be accessed by the user. The settings come pre-set for the methods used in the current paper. One accelerometry.csv file must be loaded prior to analysis. **(B)** Tremor App GUI after successful loading of.csv file, showing the file details (dashed box). The analysis routine is automatically performed as per the Methods section following activation by the Run button, which prompts the user to select protocol epochs identified by light signals as a guide. Once performed, the analysis can be visualized as a summary figure (see Figure 1 and Supplementary Figure 6) or saved for later use in a MatLab structure using the Save Data button.

Supplementary Figure 4 Final tremor epochs from a full recording captured by the right arm accelerometer are presented to the user for manual validation. The protocol was repeated three times for a total of 12 active positions (three for each posture) with 12 intervening rest epochs of 10 s each. These selected epochs are then used to calculate and present the corresponding acceleration power spectra.

Supplementary Figure 5 Comparison of accelerometry results in the ET group vs. controls for the dominant arm. In ET, the total power increases in different postural conditions compared to rest, with the highest average power noted in this case in the Wing and Action conditions. The ET population shows significant variability between patients in both total power and peak power of acceleration (error bars, SEM), reflecting different tremor intensities between patients.

Supplementary Figure 6 Examples of three single patients with varying CRTS (Part A) tremor scores and their corresponding acceleration power spectra. Each power spectrum is the post Fourier transform average of the three epochs comprised in one recording. Note the wide range of values of power with CRST increments, reflected in the changing *y*-axes. As patients develop higher tremor power, we observe the appearance of harmonics, which is a property often observed in oscillating systems.
