# Supplementary figures and images for: A standardized accelerometry method for characterizing tremor: Application and validation in an ageing population with postural and action tremor
Source: Front Neuroinform. 2022 Aug 4;16:878279. doi: 10.3389/fninf.2022.878279 (PMC9386269; doi:10.3389/fninf.2022.878279)

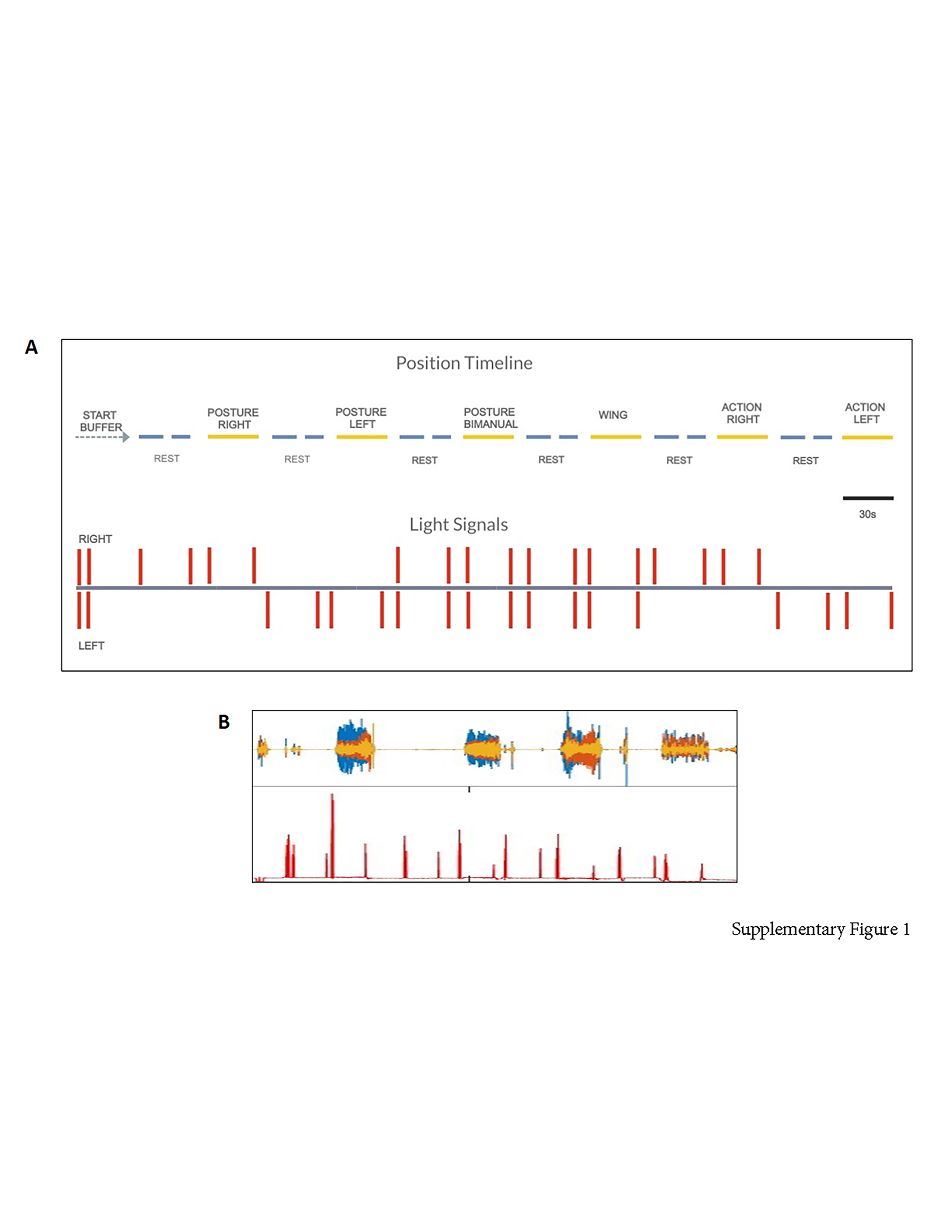

Supplement: Supplementary Figure 1 — (A) Timeline of standardized protocol used to record tremor in different upper limb positions during wristwatch based accelerometry. Data acquisition is performed sequentially at rest, then with with one arm extended (Posture Unimanual), followed by extension of the contralateral arm, bimanual arm extension (Posture Bimanual), bimanual wing position (Wing), and finally with either arm moving with a to and fro drinking motion whilst holding a 500 mL water filled plastic bottle (Action). One or two signals were applied to activate the light sensor on the wristwatch, signaling the start or end of different standardized postural conditions. (B) Resulting raw data from the acquisition. Blue, yellow, and orange traces represent detrended x,y,z accelerometry data. Red trace represents luminosity (lux), used as an event marker. [file Image_1.tiff]

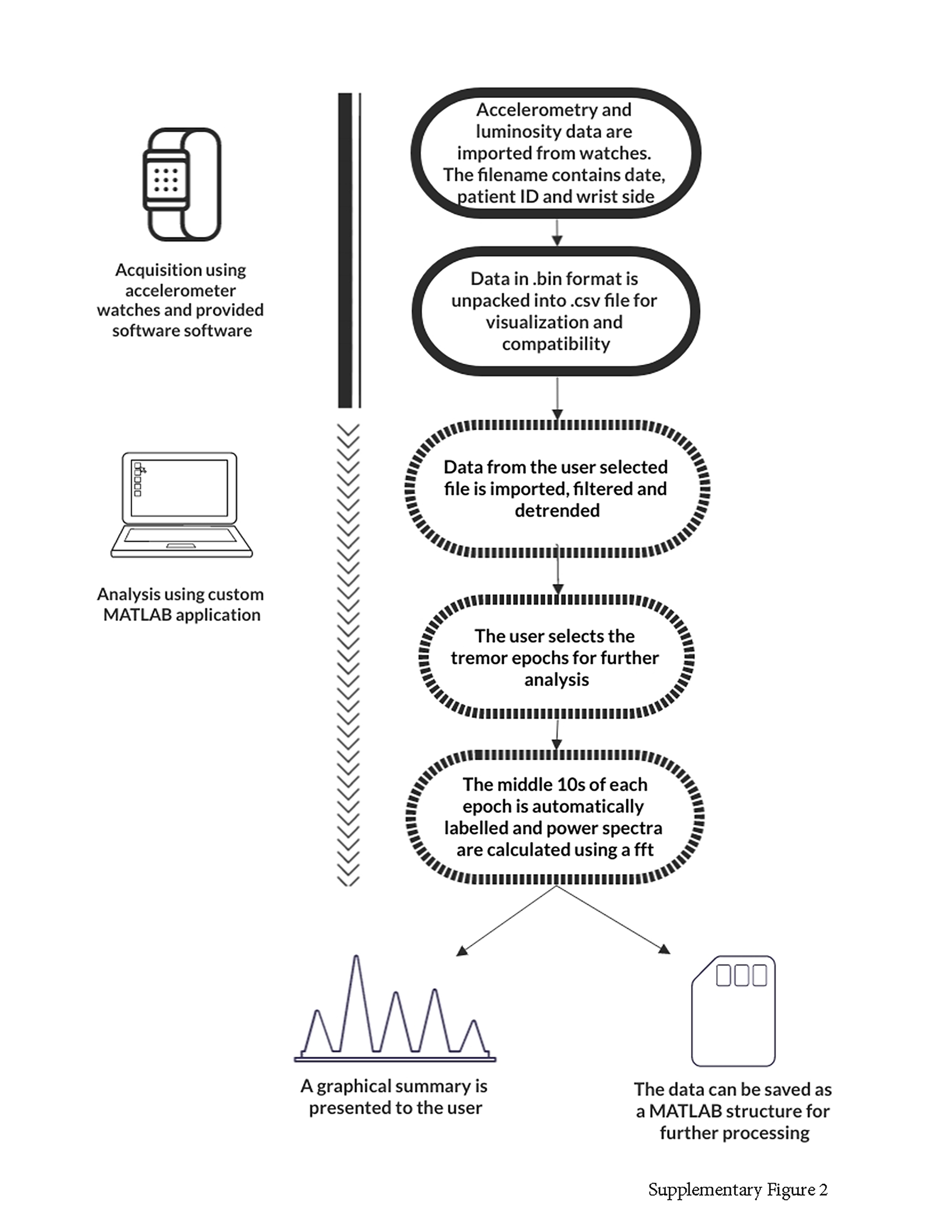

Supplement: Supplementary Figure 2 — Conceptual diagram of the analysis workflow from data acquisition (solid vertical line and bounding boxes) to analysis (chevron-pattern vertical line and interrupted boxes). Watch logo represents steps using GeneActiv© accelerometer watches and accompanying software. Laptop logo represents steps using the customized application created in MatLab, designed for tremor analysis (see Supplementary Figure 3). [file Image_2.tiff]

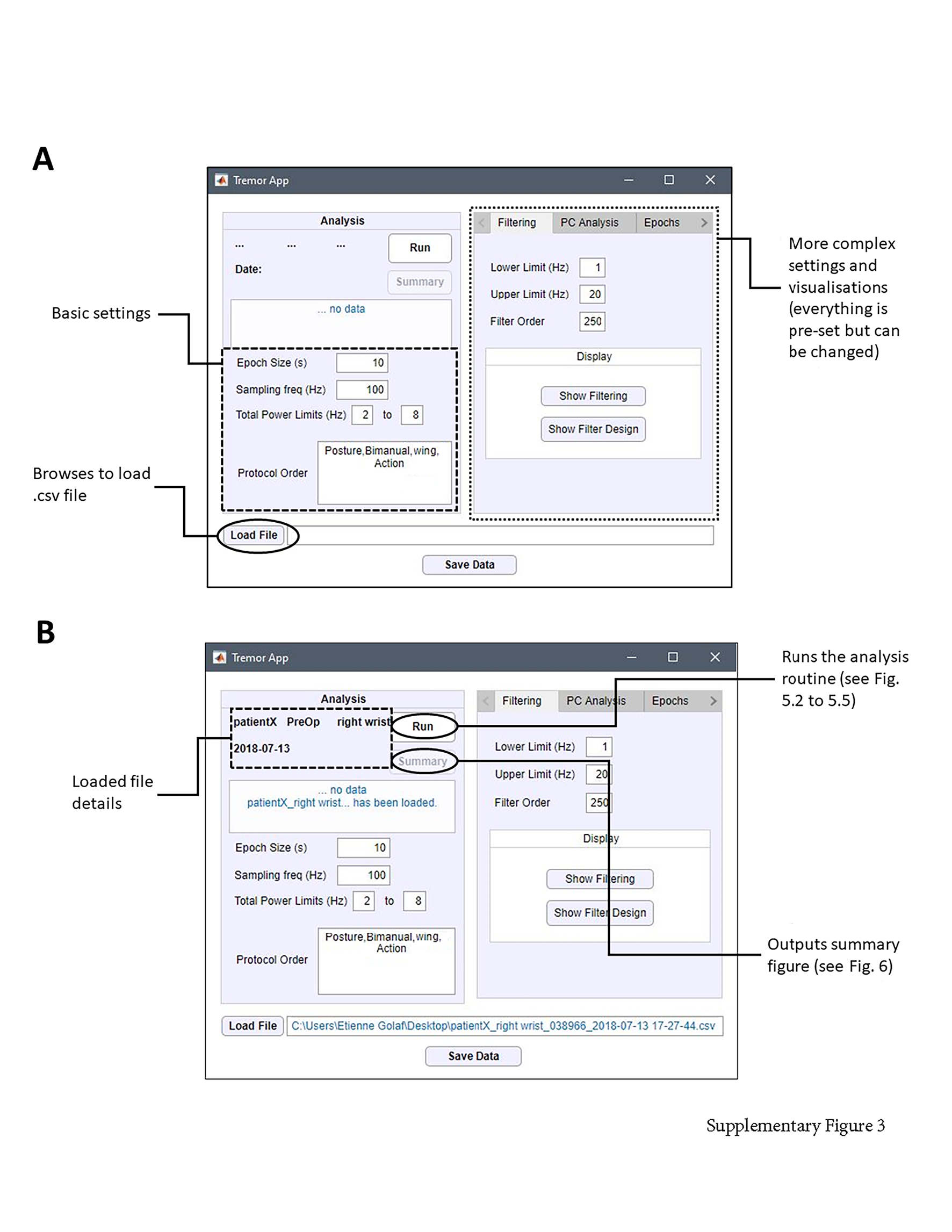

Supplement: Supplementary Figure 3 — (A) Graphical user interface (GUI) visualized upon launch of the MatLab application (Tremor App). Basic analysis settings (dashed box) can be verified and changed. Advanced settings (dotted box) including features such as filter design and principal component visuals can also be accessed by the user. The settings come pre-set for the methods used in the current paper. One accelerometry.csv file must be loaded prior to analysis. (B) Tremor App GUI after successful loading of.csv file, showing the file details (dashed box). The analysis routine is automatically performed as per the Methods section following activation by the Run button, which prompts the user to select protocol epochs identified by light signals as a guide. Once performed, the analysis can be visualized as a summary figure (see Figure 1 and Supplementary Figure 6) or saved for later use in a MatLab structure using the Save Data button. [file Image_3.tiff]

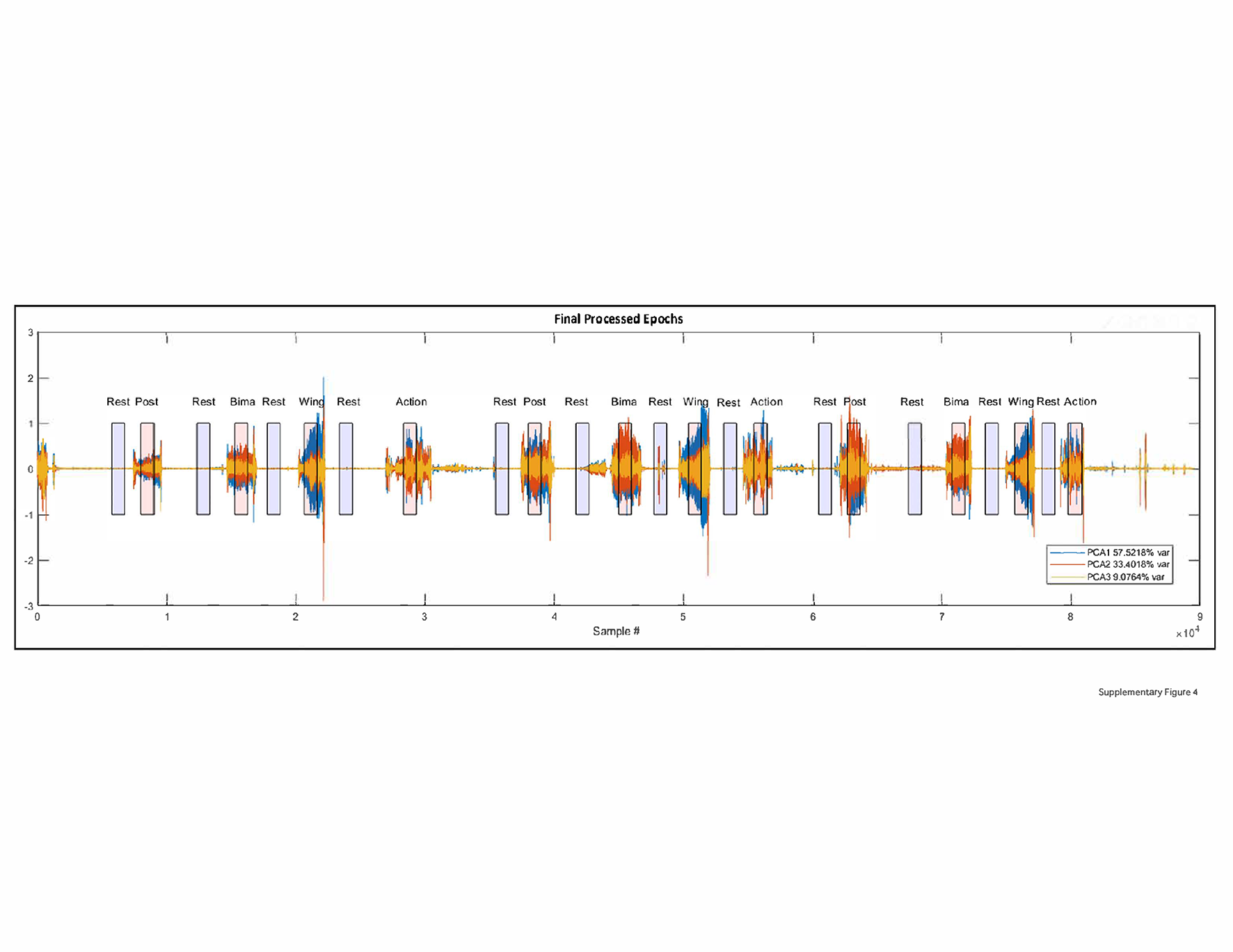

Supplement: Supplementary Figure 4 — Final tremor epochs from a full recording captured by the right arm accelerometer are presented to the user for manual validation. The protocol was repeated three times for a total of 12 active positions (three for each posture) with 12 intervening rest epochs of 10 s each. These selected epochs are then used to calculate and present the corresponding acceleration power spectra. [file Image_4.tiff]

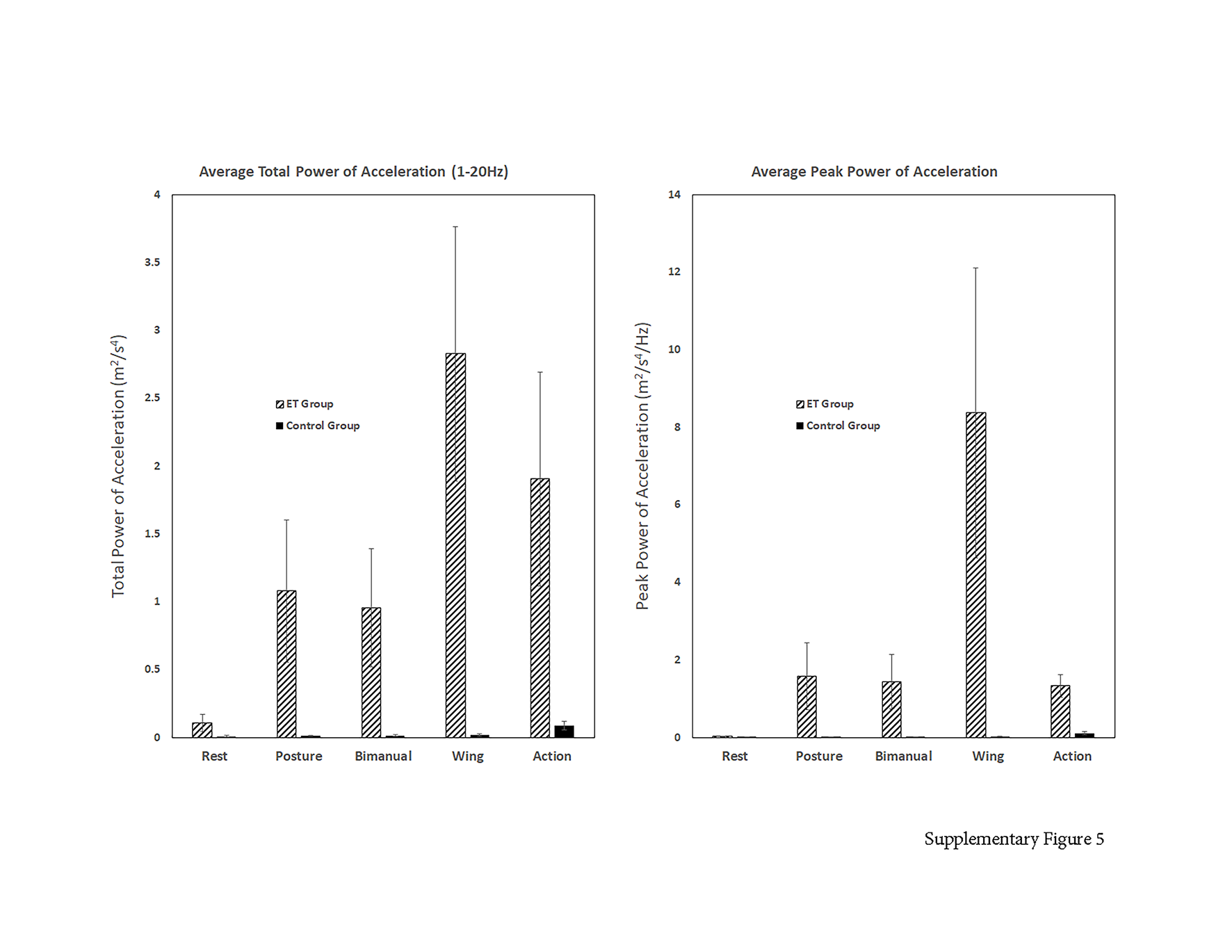

Supplement: Supplementary Figure 5 — Comparison of accelerometry results in the ET group vs. controls for the dominant arm. In ET, the total power increases in different postural conditions compared to rest, with the highest average power noted in this case in the Wing and Action conditions. The ET population shows significant variability between patients in both total power and peak power of acceleration (error bars, SEM), reflecting different tremor intensities between patients. [file Image_5.tiff]

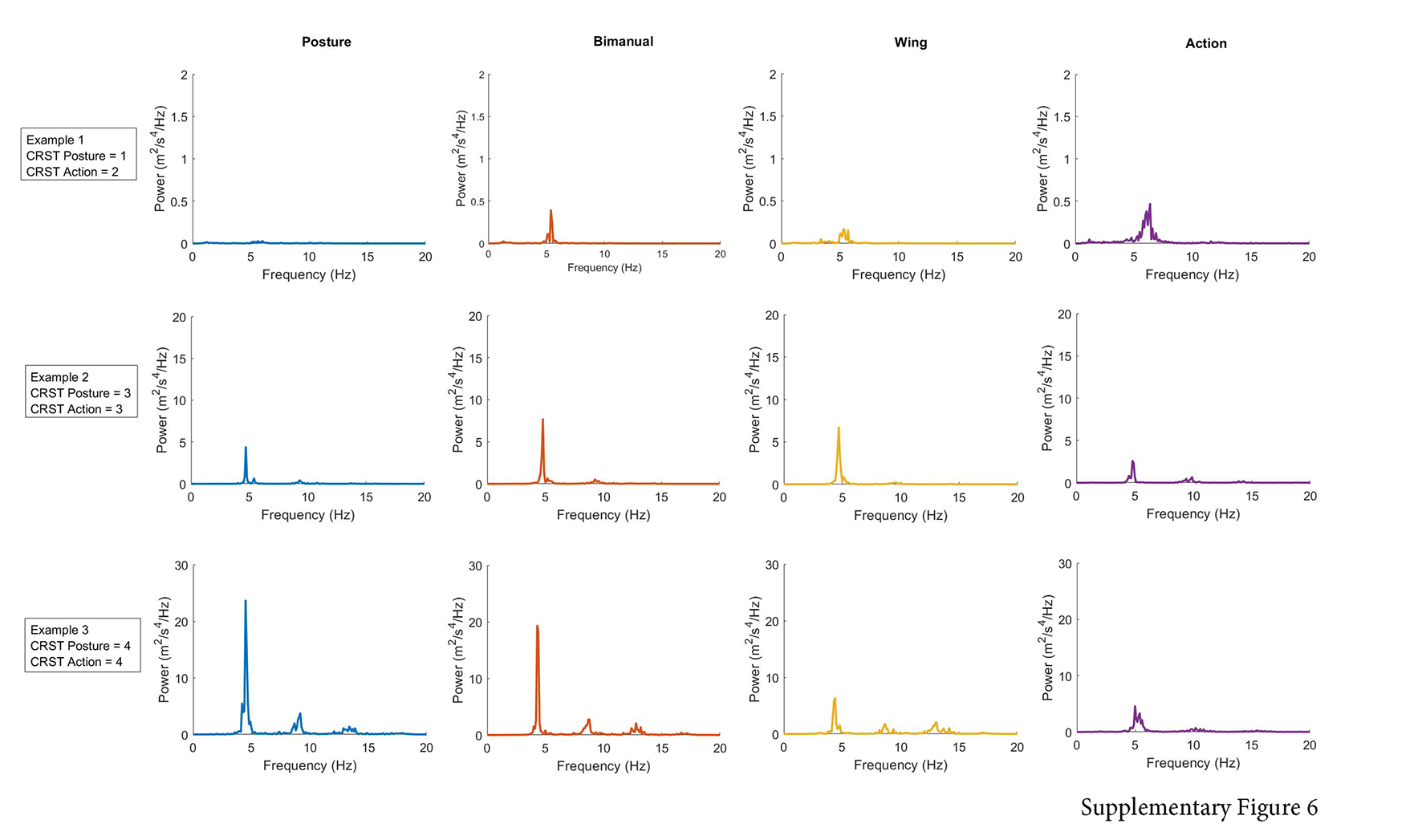

Supplement: Supplementary Figure 6 — Examples of three single patients with varying CRTS (Part A) tremor scores and their corresponding acceleration power spectra. Each power spectrum is the post Fourier transform average of the three epochs comprised in one recording. Note the wide range of values of power with CRST increments, reflected in the changing y-axes. As patients develop higher tremor power, we observe the appearance of harmonics, which is a property often observed in oscillating systems. [file Image_6.tiff]
