# Supplementary material for: A standardized accelerometry method for characterizing tremor: Application and validation in an ageing population with postural and action tremor
Source: Front Neuroinform. 2022 Aug 4;16:878279. doi: 10.3389/fninf.2022.878279 (PMC9386269; doi:10.3389/fninf.2022.878279)
Supplement: Supplementary file 7 [file Data_Sheet_1.PDF]

Supplementary Section

The supplementary section describes the method developed for tremor analysis using data from the structured protocol from a single recording session. This includes acquisition of raw data, coding and filtering, sorting of relevant epochs used to generate power spectra, principal component analysis of the acceleration signal, Fourier analysis of the acceleration signal used to generate the power spectrum of tremor, and other applied mathematics. The section also includes supplementary figures that illustrate the analysis, present the software platform, and provide a data sharing plan.

Accelerometry Analysis

1. Data acquisition

The accelerometer data is downloaded in raw .bin format from the wristwatches. The data is first converted to .csv format using the software provided with the wristwatches (GENEActiv©). These .csv files can be viewed by the user and subsequently analyzed with the MATLAB application. A conceptual diagram illustrates the workflow from data-acquisition to analysis in MATLAB (Supplementary Figure 2). Relevant screenshots of the app that was developed specifically for analysis of these files using a graphical user interface (GUI) format that allows efficient file analysis by users who may not be comfortable in MATLAB programming are presented in Supplementary Figure 2.

2. Data filtering

The raw data is composed of 3 vectors: x, y, and z accelerations (units are recorded in Gs). These signals are at first bandpass filtered from 1Hz to 20Hz using a finite impulse response filter of the 250th order, after which a simple linear detrending is performed to remove any first order shift. This eliminates most of the noise associated with voluntary motion (low frequency) and reduces high frequency random noise, keeping a conservatively large bandwidth that captures all known tremor frequencies.[15]

3. Data coding and sorting of epochs

Once the data is filtered, the app user is prompted with the full recording sample and is assisted with manual selection of the protocol period based on the acceleration tracing and the light cues. A principal component analysis is then performed which re-organizes the x,y,z accelerometry traces into three principal components in decreasing order of component variance. The user is then presented with the resulting principal components superimposed on a time series graph. Each period corresponds to the participant's different positions (Supplementary Figure 1) that are then manually selected using light cues as a guide, and automatically named. The middle 10 seconds of each period is then automatically saved for the tremor analysis to eliminate margin artefacts. These epochs are labelled, organized, and saved for further processing. The resulting periods are presented to the user for visual validation (Supplementary Figure 4).

4. Spectral analysis

Once the epochs are labelled, the first principal component is used to compute the power spectrum. Here, the principal components consist of three orthogonal vectors in 3-space, each representing an acceleration signal. The principal components are calculated such that the first component contains the largest possible variance, thus containing most of the accelerometry signal. The first principal component is used in these results for three reasons. First to capture the tremor in its main direction of oscillation, second to eliminate orthogonal oscillations from the analysis, which most likely represent noise, and third to reduce computational load. A Discrete Fourier Transform is applied to each 10 second epoch (1001 data points at 100Hz) for a specific position assumed by the participant during the structured protocol, providing a one-sided power spectrum.

In MATLAB the Fast Fourier Transform (*fft*) function performs a discrete Fourier transform from a vector as:

$$Y(k) = \sum_{j=1}^{n} X(j) W_n^{(j-1)(k-1)} \quad (1)$$

where, $$W_n = e^{(-2\pi i)/n} \quad (2)$$

The single sided power spectrum is then computed by squaring the amplitude spectrum as

$$P(k) = \frac{|Y|^2}{n} \quad (3)$$

where *n* is the number of samples, *Y(k)* is a vector representing the amplitude spectrum (in this case acceleration with units = G) for frequencies *k* (units = Hz), and *P(k)* is a vector representing the single sided power spectrum of acceleration.

The acceleration power spectrums of the position-specific epochs are averaged, and the resulting curve is used to derive 3 measures: 1) the peak power of acceleration, defined as the point of maximum intensity on the acceleration power spectrum; 2) the peak frequency, defined as the frequency at peak power; and 3) the total power of acceleration. Mathematically total power is the area under the curve of the power spectrum. In this paper the total power within the 1-20Hz frequency band (simple integral from 1Hz to 20Hz) is used. To approximate the integral of the power spectrum in these discrete datasets, the trapezoidal numerical integration method is applied. In MATLAB, the function *trapz* performs trapezoidal numerical integration as

$$\int_a^b f(x)dx = \frac{b-a}{2N}\left[f(x_1) + 2f(x_2) + \cdots + 2f(x_N) + f(x_{N+1})\right] \quad (4)$$

where the spacing between each point is equal to scalar value $\frac{b-a}{N}$ and N is the number of discrete data points.

Data Sharing

The MATLAB library and scripts used in the present study will be made available to readers using a public repository (e.g., PhysioNet). The raw and pre-processed patient data that support the findings of this study will be available on request from the corresponding author.
